# Marginalized, but not demobilized: Ethnic minority protest activity when facing discrimination

**DOI:** 10.1177/01925121231156633

**Published:** 2023-09-30

**Authors:** Antoine Bilodeau, Stephen E White, Clayton Ma, Luc Turgeon, Ailsa Henderson

**Affiliations:** Department of Political Science, Concordia University, Canada; Department of Political Science, Carleton University, Canada; Department of Political Science, Concordia University, Canada; School of Political Studies, University of Ottawa, Canada; School of Social and Political Science, University of Edinburgh, UK

**Keywords:** Protest politics, ethnic minorities, discrimination, Canada, ethnic networks, political participation, intragroup contacts

## Abstract

In a context of backlash against diversity in many countries, we know little about how ethnic minorities respond politically when they personally experience discrimination. Moving beyond the study of electoral participation, this research investigates whether experiences of discrimination push ethnic minorities toward an alternate political pathway for those who feel sidelined by the political community: protest activity. The study also examines whether the context of discrimination (i.e. public or private sphere) has different consequences for protest participation, and whether intragroup contact enhances the effects of discrimination on protest participation. Relying on a survey of 1647 respondents from racialized backgrounds in Canada, our findings indicate that discriminatory experiences increase participation in protest activities irrespective of its context, and that the positive relationship between discriminatory experiences and protest activity is stronger among respondents with greater intragroup contact.

This study investigates the effects of discriminatory experiences on ethnic minorities’ participation in protest activities. Such an investigation appears timely in a context in which the politics of immigration in western democracies since the 1990s has been characterized by a backlash against notions of diversity and multiculturalism. Although this backlash is perhaps most clearly expressed as negative discourses and more restrictive policies with respect to immigration and ethnocultural diversity, it also takes the form of discrimination against individual members of ethnic minority groups. Discrimination against ethnic minorities predates the current period, but ethnic minorities today face renewed attacks which seek to undermine their place within the national community. Although the backlash against diversity is increasingly salient, we know little about how ethnic minorities at the center of these debates react when they are the individual victims of discrimination.^1^

To address this matter, we assess the relationship between reported experiences of discrimination and participation in such protest activities as taking part in demonstrations, signing petitions or joining boycotts. Even though protest activities have been normalized over the last half-century (Dalton, 1996; see also Barnes et al., 1979; Inglehart, 1990; Nevitte, 1996; Norris, 2002), historically they have also been viewed as the preferred channel for the marginalized strata of society to voice their demands (Van Aelst and Walgrave, 2001). Accordingly, these activities, often taking place at the margins of the political system, might still represent a crucial alternate pathway to political engagement for people sidelined by the broader political community. Participation in protest activities is also said to be preferred by ‘critical citizens’ (Norris, 2002) – people who prefer to rely less on representatives to speak on their behalf, and who instead seek more direct empowerment. For ethnic minorities experiencing discrimination, protest might represent an alternative to mainstream political activity and constitute a means by which they can take matters into their own hands.

We are not the first to examine how discrimination relates to political participation. With regard to protest (and other non-electoral) activities, a few studies document greater political engagement among those reporting experiences of discrimination. In the United Kingdom, Heath et al. (2013) observe that ethnic minorities reporting discrimination are more likely to engage in protest activities, a finding replicated by Martin (2016) for the specific case of British Muslims. In the United States, Wrinkle et al. (1996) observe that discrimination is associated with greater non-electoral participation among Mexican Americans and Puerto Ricans, a relationship also observed among Latinos (Valdez, 2011).^2^ Examining racialized minorities in Quebec, Canada, Bilodeau (2017) notes a similar positive relationship between discrimination and participation in activities such as boycotts, petitions or demonstrations in the streets. Similarly, Reitz and Banerjee (2007) observe that perceived discrimination is associated with a greater propensity to volunteer for social organizations in Canada. The evidence, then, is that ethnic minorities have a significant capacity to mobilize for political action to defend their place within the community. Discrimination is a negative experience for ethnic minorities, but one that seems to instill a greater sense of urgency to political action.^3^

Building on this accumulated but sparse evidence, this study makes two contributions. First, almost no studies on this topic differentiate between different contexts in which discrimination takes place, and implicitly assume that all discriminatory experiences have the same consequences for political participation. Recently, however, Oskooii (2016, 2020) proposed that not all forms of discrimination lead to the same consequences for political participation, and demonstrated that although *political discrimination* increases political participation, *societal discrimination* actually decreases participation. We re-examine the proposition that the context in which discrimination occurs has distinctive effects on political participation, looking specifically at the case of protest activities.

Second, this study improves our understanding of the conditions under which discrimination leads ethnic minorities to become active in protest activities. More specifically, it examines whether intragroup contact can enhance the effect of discrimination on political participation by helping to transform the motivation to act into concrete action. Ethnic networks and intragroup contact are often thought to give central support to ethnic minorities by providing, among other things, social and cultural capital (Portes et al., 2009), as well as by serving as mobilizing agents for political action (Berger et al., 2007; Bilodeau, 2009; Martin, 2016). Likewise, ethnic networks and intragroup contact could play a key facilitative role in the political participation of ethnic minorities experiencing discrimination by mobilizing people with shared experiences and hence by effectively targeting and problematizing the issue of discrimination faced by the community.

The study examines the case of Canada. Decades of sustained and relatively high levels of immigration have transformed the country’s demographic profile, and Canada prides itself on its policy of multiculturalism. However, discrimination is still prevalent in the country (Godley, 2018), and few Canadian studies examine how this discrimination affects the political participation of Canadians from diverse backgrounds. We explore the relationship between discrimination and protest participation by investigating the specific case of racialized Canadians, relying on a survey of 1647 respondents from ‘visible minority’ backgrounds in Quebec, Ontario, Alberta and British Columbia, the four provinces with the largest number of racialized people.

As per Statistics Canada’s (2011) definition, ‘visible minorities’ are people, other than indigenous peoples, who are non-Caucasian in race or non-white in color. In 1986, the federal government of Canada formally introduced the category of visible minorities as part of the Employment Equity Act to document and address the systemic discrimination experienced by members of these communities (Statistics Canada, 2011). Canada’s visible minority population has increased significantly since the 1960s. In 1981, the first year for which we have data, members of visible minorities made up 4.7% of the Canadian population (Statistics Canada, 2011); by 2016, the proportion was 22.3% (Statistics Canada, 2017).

Studies demonstrate that visible minorities in Canada are today still more likely than the rest of the population to experience social and economic marginalization (Nangia, 2013; Reitz and Banerjee, 2007). It is also well documented that people of visible minority background exhibit lower levels of political participation than the rest of the population, with most studies documenting the gap in electoral participation (Bilodeau and Turgeon, 2015; Reitz and Banerjee, 2007; Tossutti, 2007). We propose to expand the study of the relationship between discrimination and political participation in protest activities, with an aim to improve our understanding of how racialized Canadians respond politically to discrimination.

## Discrimination in public and private spheres

Nearly all existing studies on the link between discrimination and political activity treat discrimination as a generalized experience. Some use dichotomous measures indicating whether respondents report having experienced discrimination or not; others use scales measuring the frequency of such experiences. Rarely have scholars considered the context in which discrimination occurs. One approach to addressing the matter of when and where discrimination occurs is to look at whether the relationship between discrimination and political participation or belonging is the same across political communities (Bilodeau et al., 2022; Gaasendam, 2022), but another approach is to examine the micro context in which discrimination takes place. Taking the latter approach, Oskooii (2016, 2020) distinguishes between *political discrimination*, measured by individuals’ reports of discrimination when dealing with, for example, government officials, or the police and courts, and *societal discrimination*, measured by individuals’ reports of unfair treatment at social gatherings, on the street or in shops, banks, restaurants or bars.

Oskooii (2020) argues that political discrimination and societal discrimination may not have the same consequences for political participation; although the former has the ‘capacity to make politics more salient, motivating individuals to act collectively against institutions or actors that violate notions of equality and fairness embedded within democratic regimes’ (868), the latter likely undermines the desire and capacity of individuals to participate in the political process by making them feel rejected by the broader community. However, both political or societal discrimination, by signaling an exclusion from the broader society, likely reinforce the ethnic identity and hence increase participation in ethnic activities. Analyzing data from two distinct contexts, the United Kingdom (2020) and among Muslims in the United States (2016), Oskooii finds that political discrimination encourages participation in a broad range of social and political activities, but societal discrimination appears to depress mainstream political participation in activities such as voting.

Building on Oskooii’s important contribution, we also investigate how the context in which discrimination takes place may have different ramifications for ethnic minorities’ political participation. We differentiate between discriminatory experiences that take place in the public sphere and those that take place in the private sphere. We define *public sphere discrimination* as experiences of discrimination that take place in relation to agents of the state such as police officers, judges or any representative of the state. We define *private sphere discrimination* as experiences of discrimination that take place in relation to private citizens, such as in restaurants, in looking for work or housing or in relations with neighbors.

Moreover, we examine more closely the effect of discrimination on protest activities, forms of political action only briefly examined by Oskooii. Oskooii (2020) argues that mainstream routes to political participation may not appeal to ethnic minorities experiencing societal discrimination, and that ethnic activities are more attractive channels to express their voices (869). We agree with Oskooii, and we do not think this positive effect is limited to ethnic activities. As mentioned, in sharp contrast to voting and other electoral activities, protest participation often occurs at the margins of the political system and hence may be just as appealing as ethnic activities for individuals who experience discrimination. From this perspective, we expect both public sphere and private sphere discrimination to be motivating factors leading to speak up in protest activities (Hypothesis 1).^4^

## Intragroup contact as enhancers for the discrimination effect

How much of a role does intragroup contact play in the mobilization process for protest activities? Considering that we know people tend to participate in politics when they are asked to do so (Verba et al., 1995) and that many protest activities are often collective endeavors as opposed to individual ones (Van Deth, 2014), we expect visible minorities to be more likely to engage in protest activities when they are connected to other people, irrespective of whether those other people qualify as ‘co-ethnics’. The specific question that interests us, however, concerns the role of intragroup contact in supporting political mobilization when facing discrimination.

Beyond testing the distinctive effects of public sphere and private sphere discrimination, we also examine the role of intragroup contact in moderating the effects of discrimination on participation in protest activities. We know that ethnic networks and intragroup contact play a crucial role in the social and political lives of members of ethnic communities. A number of studies indicate a relationship between the presence of ethnic networks or intragroup contact and greater political participation among ethnic minorities (Berger et al., 2007; Bilodeau, 2009; Martin, 2016), either because they mobilize ethnic minorities (Fennema and Tillie, 1999; Tillie, 2004; Uhlaner, 1989) or because they provide a feeling of empowerment (Leighley, 2001).

If ethnic networks and intragroup contact can facilitate political participation through mobilization efforts or by creating a feeling of empowerment, we might expect their effectiveness to be enhanced when mobilizing individuals who hold grievances against the broader community. By being in contact with people who have either faced similar discrimination or who have empathy because they could at least easily face similar discrimination, mobilization would help such individuals to take action to seek reparations or work toward social change. Although discrimination would provide the impetus for political action, intragroup contact would facilitate the transformation of the motivation to participate into effective political action. Put differently, because of discrimination, ethnic minorities might wish to become politically active, but mobilizing for political action might be more challenging if one feels alone in experiencing discrimination. However, when ethnic minorities experience discrimination and are in frequent contact with people of the same ethnic background, mobilization for political action might be easier. Accordingly, the combination of discrimination and the presence of a strong intragroup contact together would facilitate the political participation of ethnic minorities, more so than if only one of these two conditions is present.

We expect that having frequent contact with people of the same ethnic background will amplify the effect of discrimination in increasing participation in protest activities. More specifically, we expect ethnic minorities experiencing (public or private sphere) discrimination to be more active in protest activities than those not experiencing discrimination, and ethnic minorities experiencing (public or private sphere) discrimination and in frequent contact with people of the same ethnic background as themselves to be even more politically active in protest activities (Hypothesis 2).

## Research design and data

The study relies on data derived from the *Provincial Diversity Project* (PDP), a 25-minute online survey conducted during the winter of 2014, which includes a special stratified sample of 1647 respondents of visible minority background living in 4 provinces (Quebec, Ontario, Alberta and British Columbia).^5^ At times, we also rely on the general population component of the PDP to offer points of comparison of how white Canadians behave in comparison to the visible minority population. The sample of white Canadians for the 4 provinces examined is 3350 respondents. Our comparison with white Canadians, however, is limited to descriptive data for participation in protests; questions on discrimination were given only to visible minority respondents, thus limiting our capacity to compare the role of discrimination on the political participation of non-visible minority Canadians.

Existing studies rely on two distinct strategies to measure discrimination. One focuses on personal experiences of discrimination (e.g. Bilodeau, 2017; Heath et al., 2013; Valdez, 2011; Wrinkle et al., 1996) whereas the other focuses on individual perceptions that their own ethnic group is a victim of discrimination in the community (e.g. Chong and Rogers, 2005; Heath et al., 2013; Maxwell, 2006; Oskooii, 2016). In line with the first approach, our study measures ethnic minorities’ personal experiences of discrimination. In our opinion, such a measurement approach is more likely to reflect lived experiences of discrimination, whereas respondents’ perceptions of group discrimination are more likely to capture a general attitude rather than lived experiences.^6^

Respondents were asked how often, in the last five years, they had experienced discrimination because of their race, ethnicity, or religion when dealing with each of the following: (a) landlords; (b) co-workers or bosses; (c) neighbors; (d) government employees; and (e) the police. Respondents were given the following options: several times, a few isolated incidents, never.^7^ Table 1 presents the proportions of visible minority respondents reporting discriminatory experiences in each of the five contexts. Visible minorities report discrimination most frequently in relation to the workplace (with 43% reporting isolated or several incidents) followed by interactions with government employees (34%); discriminatory experiences appear least frequent in relation to landlords (23%).

**Table 1.** Experiences of discrimination in five domains of life.

Discrimination by . . .	Never (%)	Isolated incidents (%)	Several incidents (%)	Number of observations
**Landlords (%)**	77	14	9	1563
**Co-workers or bosses (%)**	57	28	15	1556
**Neighbors (%)**	68	21	11	1545
**The police (%)**	72	16	12	1548
**Government employees (%)**	66	22	12	1519

Source: 2014 Provincial Diversity Project.

We combined the indicators for discrimination by government employees and the police to form the *public sphere discrimination* index, and we combined those for discrimination by landlords, neighbors and co-workers and bosses to form the *private sphere discrimination* index. Both measures range from 0 to 10, where 10 indicates frequent discrimination; the mean scores are respectively 2.1 and 2.3. About 40% of visible minority respondents report having been discriminated against at least once in the public domain; the proportion is 62% for the private domain.^8^ In addition to these measures of discrimination, we also created an index that combines all five indicators, merging both public sphere and private sphere discrimination; the mean score for the index is 2.2. We compare both models of participation in protest activities (using the distinctive indices of public sphere and private sphere discrimination vs. using the full-discrimination index).

We examine three indicators of participation in protest activities. Respondents were asked to indicate if they had participated in each of the following activities over the last 12 months: signing a petition, joining a boycott and joining a demonstration. Table 2 indicates that, within the last 12 months, visible minority Canadians are less likely than white Canadians to have joined a boycott (22% vs. 33%) or to have signed a petition (34% vs. 48%); in contrast, visible minority Canadians appear somewhat more likely to have taken part in a street demonstration (10% vs. 7%). Finally, we combine all three indicators of participation in a multi-item index ranging from 0 to 1 where 1 means that respondents have engaged in all three forms of participation, and 0 means they have not engaged in any of the three forms of participation. The mean score for visible minority Canadians in the sample (.22) is somewhat lower than that for other Canadians (.30).

**Table 2.** Participation in protest activities.

	Visible minority Canadians	White Canadians
**Joined a boycott (%)**	22	33
**Signed a petition (%)**	34	48
**Demonstrated in the street (%)**	10	7
**Participation score (0–1, mean)**	.22	.30
**Number of observations**	1545	3278

Source: 2014 Provincial Diversity Project.

In order to verify the role of intragroup contact in facilitating political mobilization among those experiencing discrimination, we rely on an indicator that asked visible minority respondents how often they spend time with people of the same ethnic group as themselves – aside from their families (every week, once or twice per month, a few times per year or never). About 20% of visible minorities in our sample report having contact with people of their own ethnic group every week, 29% report having such contact once or twice per month, 34% only a few times per year, and 16% report never having contact with people of their own ethnic group. The following sections verify our hypotheses using multivariate analyses.

## Discrimination and participation in protest activities

To assess the relationship between (public sphere and private sphere) discrimination and participation in protest activities, we conduct two sets of multivariate analyses; the first examines the effect of the combined index discrimination, and the second distinguishes public sphere and private sphere discrimination. These multivariate analyses include socio-demographic control variables that are known predictors of engagement in protest activities: age, education, unemployment status, household income and sex (Dalton, 2008) as well as foreign-born status (Bilodeau, 2008). We also include province of residence because it is known to be a significant predictor of political attitudes and behaviors among ethnic minorities in Canada (Bilodeau et al., 2015). Additionally, because different minority groups might report different experiences with discrimination, we consider respondents’ ethnic background. Likewise, because Muslims have been subject to significant media attention and public backlash, we include a variable indicating whether respondents are Muslim or not. Finally, we also include measures of interest in politics, general social contact and intragroup contact, key variables in predicting levels of participation (Verba et al., 1995). Our analyses include only respondents who are members of a visible minority group because the questions about discrimination were not given to white respondents.

Table 3 reports the results for the analyses testing the combined index of discrimination. The analyses indicate that immigrants are less likely than non-immigrants to participate in boycotts and sign petitions and that women are less likely to sign petitions and participate in public demonstrations. We observe no systematic differences between different ethnic minority groups. As seen in Table 2, discernible differences seem to emerge between visible minority Canadians and white Canadians rather than between different ethnic minorities. Finally, respondents with denser social networks appear more likely to engage in boycotts and public demonstrations, but the analyses reveal no significant differences between visible minority respondents who have frequent contact with co-ethnics and those who do not.

**Table 3.** Discrimination and participation in protest activities.

	Joined a boycott^1^		Signed a petition^1^		Attended a demonstration^1^		Participation index (0–1)^2^	
	*B*	*SE*	*B*	*SE*	*B*	*SE*	*B*	*SE*
Age	.00	(.01)	-.01	(.01)	-.01	(.01)	-.00	(.00)
Immigrant	-.67**	(.21)	-.43*	(.18)	-.36	(.30)	-.08**	(.02)
Education (ref. cat. High School)								
Postsecondary	-.23	(.38)	-.12	(.31)	-.91*	(.45)	-.06	(.05)
Undergraduate university	.03	(.35)	-.07	(.30)	-.77	(.42)	-.04	(.04)
Postgraduate university	-.04	(.39)	.20	(.32)	-.72	(.47)	-.01	(.05)
Female	-.26	(.20)	-.40*	(.16)	-.80**	(.28)	-.06**	(.02)
Unemployed	.04	(.40)	.09	(.38)	.78	(.44)	.03	(.04)
Province (ref. cat. Ontario)								
Quebec	.03	(.23)	.49*	(.20)	.41	(.33)	.04	(.03)
Alberta	-.17	(.24)	-.14	(.20)	-.23	(.38)	-.02	(.02)
British Columbia	-.15	(.23)	.37	(.20)	.12	(.35)	.03	(.02)
Ethnic origin (ref. cat. other)								
Black	-.08	(.29)	.13	(.25)	-.12	(.40)	.01	(.04)
Chinese	-.54*	(.25)	-.36	(.21)	-.51	(.39)	-.06*	(.02)
South East Asia	-.52	(.27)	-.16	(.24)	-.25	(.40)	-.05	(.03)
Muslim	.46	(.30)	-.06	(.26)	.87*	(.40)	.06	(.04)
Interest in politics	.15***	(.04)	.14***	(.03)	.05	(.05)	.02***	(.00)
General social network	1.41**	(.48)	.75	(.43)	2.29***	(.69)	.18***	(.05)
Intragroup contact	.01	(.12)	.08	(.10)	.21	(.16)	.01	(.01)
Discrimination (0–10)	.15***	(.03)	.09**	(.03)	.28***	(.04)	.03***	(.00)
Constant	-2.58***	(.60)	-1.41**	(.50)	-3.79***	(.85)	.09	(.07)
Observations	1432		1434		1468		1458	
Pseudo/Adjusted *R*-square	.10		.08		.25		.16	

Source: 2014 Provincial Diversity Project.

1 Entries report binary logit coefficients with robust standard errors in parentheses.

2 Entries report unstandardized ordinary least squares (OLS) regression coefficients with robust standard errors in parentheses.

* *p* < .05, **p < .01, ***p < .001.

**Figure 1.**
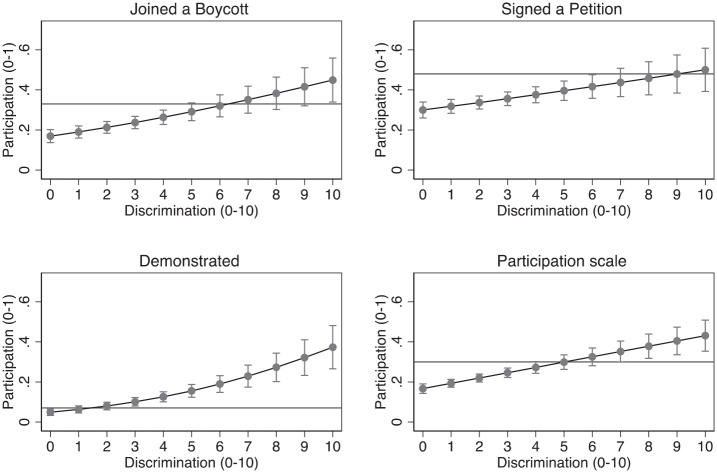
Participation in protest activities by discrimination.

In relation to the main purpose of our investigation, the analyses indicate that perceived discrimination is positively linked to all three forms of protest participation and to the multi-item index of participation, a finding largely in line with previous studies (Bilodeau, 2017; Heath et al., 2013; Martin, 2016; Valdez, 2011). As presented in Figure 1, visible minorities reporting more frequent discrimination express a greater propensity to join boycotts. The percentage of visible minorities who report engaging in this activity increases from 17% among those reporting scores of 0 on the discrimination index to 45% among those reporting scores of 10 on the discrimination index. The horizontal lines in the figure report the mean level for the non-visible minority population across the four provinces examined. As seen in the figure, visible minorities’ likelihood of joining a boycott progressively catches up with, and then surpasses, that of the rest of the population as visible minorities report more frequent experiences of discrimination in many domains of their lives.

Similarly, the percentage who report signing petitions increases from 30% to 50%, allowing visible minorities to catch up with the rest of the population for this form of protest activity. There is also a positive relationship between discrimination and the propensity to engage in public demonstrations. The relationship is strongest with respect to this activity, with the percentage of those attending public demonstrations increasing from 5% among those reporting scores of 0 on the discrimination index to 37% among those reporting scores of 10. Notably, as described in Table 2, visible minorities reported somewhat higher levels of overall engagement in demonstrations than the rest of the population (10% vs. 8%); here, we find that discriminatory experiences lead visible minorities to an almost four times greater likelihood of participating in this form of activity. Finally, the analysis for the participation index indicates that visible minorities’ participation increases with reported experiences of discrimination, and that their average level of participation catches up to that of other Canadians when their reported levels of discrimination reach the midpoint of the 0 to 10 scale. These findings suggest that when facing discrimination, ethnic minorities do not react with apathy, but rather as empowered and active citizens determined to confront discrimination.

This first set of analyses indicates a significant relationship between the combined discrimination index and participation in protest activities, but is the effect different for discrimination in public and private spheres? Table 4 reports the findings of analyses distinguishing between these two sources of discrimination. They provide mixed results regarding the impact of the source of discrimination on the participation of visible minorities in protest activities. For all three indicators of protest activities examined individually, both public sphere and private sphere discrimination predict greater engagement, although the effect is not always statistically significant. Hence, private sphere discrimination is significantly related to joining boycotts, whereas the effect of public sphere discrimination (*B* = 0.07, *p* < .10) does not reach conventional levels of statistical significance. With respect to signing petitions, neither public nor private sphere discrimination is significantly related to greater engagement, and both public sphere and private sphere discrimination significantly predict a greater propensity to participate in a demonstration. Finally, when examining the combined index of our three indicators of participation, the results indicate a positive effect of discrimination on participation when originating from both the private and the public domains. The effect is also of similar magnitude for both indices of public sphere and private sphere discrimination, with visible minority Canadians’ participation increasing by about .10 (on the 0 to 1 scale) when discrimination moves from its minimum (0) to its maximum (1) value.

**Table 4.** Public and private sphere discrimination and participation in protest activities.

	Joined a boycott^1^		Signed a petition^1^		Attended a demonstration^1^		Participation index (0–1)^2^	
Age	.00	(.01)	-.01	(.01)	-.01	(.01)	-.00	(.00)
Immigrant	-.63**	(.22)	-.38*	(.18)	-.33	(.30)	-.07**	(.03)
Education (ref. cat. Highschool)								
Postsecondary	-.20	(.39)	-.09	(.32)	-.84	(.46)	-.05	(.05)
Undergraduate university	.04	(.36)	-.05	(.30)	-.67	(.43)	-.03	(.05)
Postgraduate university	-.01	(.39)	.21	(.33)	-.63	(.47)	-.00	(.05)
Female	-.24	(.20)	-.38*	(.16)	-.80**	(.28)	-.06**	(.02)
Unemployed	.06	(.40)	.08	(.38)	.77	(.44)	.03	(.04)
Province (ref. cat. Ontario)								
Quebec	.02	(.23)	.48*	(.20)	.36	(.34)	.04	(.03)
Alberta	-.16	(.24)	-.10	(.21)	-.25	(.39)	-.02	(.02)
British Columbia	-.13	(.23)	.37	(.20)	.14	(.36)	.03	(.02)
Ethnic origin (ref. cat. other)								
Black	-.04	(.29)	.12	(.25)	-.14	(.41)	.01	(.04)
Chinese	-.50	(.26)	-.33	(.21)	-.51	(.39)	-.05*	(.02)
South East Asia	-.47	(.28)	-.11	(.24)	-.25	(.40)	-.04	(.03)
Muslim	.47	(.30)	-.07	(.27)	.86*	(.40)	.06	(.04)
Interest in politics	.15***	(.04)	.14***	(.03)	.05	(.05)	.02***	(.00)
General social network	1.41**	(.48)	.70	(.43)	2.21**	(.71)	.18***	(.05)
Intragroup contact	-.00	(.12)	.08	(.10)	.22	(.17)	.01	(.01)
Discrimination								
Private sphere (0–10)	.09*	(.04)	.04	(.04)	.16**	(.05)	.01*	(.01)
Public sphere (0–10)	.07	(.04)	.06	(.04)	.13**	(.04)	.01**	(.01)
Constant	-2.67***	(.61)	-1.43**	(.50)	-3.82***	(.85)	.08	(.07)
Observations	1408		1410		1442		1432	
Pseudo/Adjusted *R*-square	.10		.08		.25		.16	

Source: 2014 Provincial Diversity Project.

1 Entries report binary logit coefficients, with robust standard errors in parentheses.

2 Entries report unstandardized ordinary least squares (OLS) regression coefficients, with robust standard errors in parentheses.

* *p* < .05, **p < .01, ***p < .001.

At this stage, it is not clear that taking into account the unique effects of public and private sphere discrimination improves our understanding of participation in protest activities. The conclusions are similar to those when using the combined discrimination index, but less parsimonious: visible minorities reporting more frequent experiences of discrimination in different domains of their lives also report a greater propensity for protest activity. In the end, when predicting visible minorities’ participation in protest activities, the single combined index of discrimination in both public and private domains appear a more fruitful analytical strategy. Although distinguishing public and private spheres appears to help in predicting other forms of political engagement (see Oskooii, 2020), that does not appear to be the case for protest activities.

## The moderating role of intragroup contact

The final section of our analyses examines the moderating role of intragroup contact in mobilizing in protest activities ethnic minorities experiencing discrimination. Our second hypothesis states that the positive effect of discrimination on participation in protest activities is stronger for ethnic minorities in frequent contact with other people of their own ethnic background. To test the hypothesis, we perform the same analyses presented in Table 3, also adding an interaction variable between the combined discrimination index and the frequency of time spent with people of the same ethnic group. The analyses are presented in Table 5.

**Table 5.** Discrimination and participation in protest activities: The role of intragroup contact.

	Joined a boycott^1^		Signed a petition^1^		Attended a demonstration^1^		Participation index (0–1)^2^	
Age	.00	(.01)	-.01	(.01)	-.01	(.01)	-.00	(.00)
Immigrant	-.66**	(.21)	-.41*	(.18)	-.33	(.30)	-.07**	(.02)
Education (ref. cat. Highschool)								
Postsecondary	-.17	(.39)	-.06	(.32)	-.91*	(.45)	-.04	(.05)
Undergraduate university	.12	(.36)	-.01	(.30)	-.75	(.42)	-.02	(.04)
Postgraduate university	.00	(.40)	.24	(.33)	-.72	(.47)	-.00	(.05)
Female	-.29	(.20)	-.42**	(.16)	-.83**	(.28)	-.07**	(.02)
Unemployed	-.01	(.40)	.06	(.39)	.78	(.43)	.02	(.04)
Province (ref. cat. Ontario)								
Quebec	.02	(.23)	.48*	(.20)	.40	(.33)	.04	(.03)
Alberta	-.16	(.24)	-.12	(.21)	-.22	(.39)	-.02	(.02)
British Columbia	-.15	(.23)	.38	(.20)	.12	(.36)	.03	(.02)
Ethnic origin (ref. cat. other)								
Black	-.09	(.29)	.12	(.25)	-.14	(.41)	.01	(.03)
Chinese	-.51*	(.25)	-.34	(.21)	-.49	(.39)	-.05*	(.02)
South Asian	-.50	(.27)	-.15	(.24)	-.20	(.39)	-.04	(.03)
Muslim	.52	(.30)	-.03	(.26)	.90*	(.41)	.07	(.04)
Interest in politics	.15***	(.04)	.15***	(.03)	.05	(.05)	.02***	(.00)
General social network	1.25**	(.48)	.65	(.43)	2.14**	(.69)	.16**	(.05)
Intragroup contact	-.18	(.13)	-.04	(.12)	.02	(.21)	-.02	(.01)
Discrimination (0–10)	.01	(.06)	-.01	(.06)	.18*	(.08)	-.00	(.01)
Discrimination * Intragroup contact	.08*	(.03)	.06*	(.03)	.05	(.04)	.02***	(.00)
Constant	-2.24***	(.60)	-1.20*	(.50)	-3.33***	(.87)	.14*	(.07)
Observations	1432		1434		1468		1458	
Pseudo/Adjusted *R*-square	.11		.08		.25		.18	

Source: 2014 Provincial Diversity Project.

1 Entries report binary logit coefficients with robust standard errors in parentheses.

2 Entries report unstandardized ordinary least squares (OLS) coefficients, with robust standard errors in parentheses.

* *p* < .05, **p < .01, ***p < .001.

**Figure 2.**
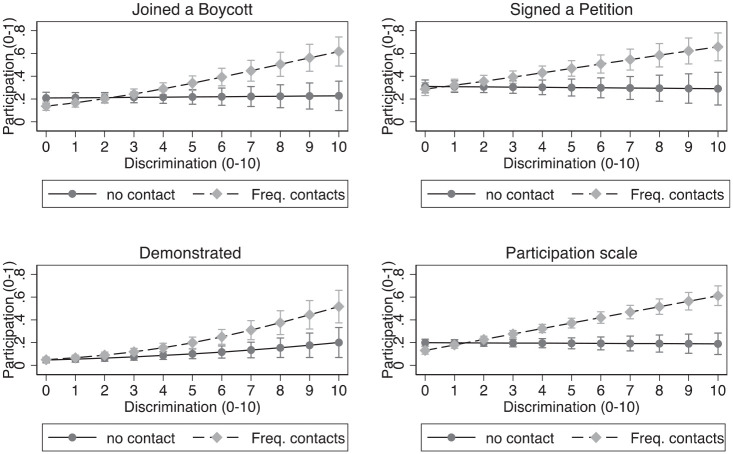
Participation in protest activities by discrimination, by intragroup contact.

The evidence supports the hypothesis regarding the moderating role of intragroup contact in facilitating participation in protest activities among members of ethnic minority groups who face discrimination. The results indicate that the greater participation of visible minorities in protest activities when experiencing discrimination is primarily observed among those who surround themselves with people of their own ethnic background on a regular basis. Figure 2 presents the predicted probabilities of engaging in each of the three protest activities for visible minority respondents who are in contact with people of their own ethnic background on a weekly basis and those who are never in contact with such people. These predicted probabilities are obtained by varying the intragroup contact and discrimination variables while keeping all other variables in the analyses at their sample mean. For all three protest activities, the mobilizing effect of discrimination is stronger when minorities spend time with people of their own ethnic background on a regular basis. As the figure indicates, when minorities never spend time with people of their own ethnic background, discrimination does not appear to be associated with a greater propensity to engage in two of the three protest activities.^9^

The results regarding participation in non-electoral political activities lend some support to the intragroup contact hypothesis. As suggested by the literature, intragroup contact appears to be an important resource in mobilizing ethnic minorities for participation in protest activities. How specifically intragroup contact increases political participation, be it either through increased awareness or just simply increased mobilization resources, remains to be seen, however, and could potentially be subject to future explorations on the subject.^10^

## Conclusion

Investigating a sample of more than 1600 racialized minorities in Canada, this study finds that discriminatory experiences increase participation in protest activities irrespective of the context, and that the positive relationship between discriminatory experiences and protest activity is stronger among respondents who have greater intragroup contact. By focusing on protest activities, an important channel through which marginalized groups can voice their demands, our study sought to examine how ethnic minorities react politically when facing exclusion.

Our findings indicate that although ethnic minorities might be marginalized by discrimination, this does not mean they are demobilized politically. Indeed, like others before us (Bilodeau, 2017; Heath et al., 2013; Martin, 2016; Wrinkle et al., 1996), we observed that ethnic minorities reporting experiences of discrimination are more likely to be involved in various forms of protest activities than those who do not report mistreatment. When discriminated against, ethnic minorities appear to seek out the same forms of political activity historically used by the marginalized strata of society.

Beyond documenting the relationship between discrimination and protest activity among ethnic minorities, our study makes two contributions. First, we wanted to verify whether recent evidence indicating that experiences of discrimination in the public and private domains have different impacts on electoral participation (Oskooii, 2016, 2020) is replicated for protest activities. When it comes to predicting the relationship between discriminatory experiences and protest activity, our investigation suggests that the outcome is the same whether the source of mistreatment is in the public or private domain: discriminatory experiences lead to a greater likelihood of participating in boycotts, petitions and public demonstrations. Distinguishing between *public sphere* and *private sphere* discrimination did not provide a better account of ethnic minorities’ participation in protest activities. When investigating the relationship between discrimination and protest participation, scholars can count on a more parsimonious model with just as much explanatory power by using an index of discriminatory experiences that combines both *public sphere* and *private sphere* discrimination. Our findings, however, do not necessarily contradict those of Oskooii. They might be explained by our specific focus on protest activities. Arguably, ethnic minorities are pushed to the margins of society when they are discriminated against in either the public or private domain; our study indicates that when that happens, ethnic minorities choose to mobilize themselves around forms of political action commonly used by the most marginalized, namely protest activities. Future research should continue to explore whether the domain in which discriminatory experiences occur affects the relationship between discrimination and involvement in other kinds of political activities.

Second, our study provides a better understanding of the conditions under which discrimination leads ethnic minorities to become active in protest activities. Existing research demonstrates that ethnic networks and intragroup contact can provide ethnic minorities with social and cultural capital (Portes et al., 2009) and serve as mobilizing agents for political action (Berger et al., 2007; Bilodeau, 2009; Martin, 2016). Our study contributes to the literature on ethnic networks and intragroup contact by demonstrating that they are essential for the protest mobilization of ethnic minorities facing discrimination. Only when ethnic minorities had strong intragroup contact do we observe that those experiencing discrimination were more active in protest activities. Our analyses indicate that when ethnic minorities are completely isolated from contact with co-ethnics, experiences of discrimination do not lead to greater participation in protest activities. Demonstrating the specific mechanism at work is beyond the scope of our study, but one possibility is that intragroup contact facilitates the political participation of ethnic minorities experiencing discrimination by mobilizing people with shared experiences, and by effectively targeting and problematizing the issue of discrimination faced by the community. We leave the formalization of the mechanism for future researchers, but it appears that intragroup contact helps to transform the motivation to act into concrete action.

In conclusion, it appears important to underline that like most other studies on the topic of discrimination and political activity, we presume that it is primarily discrimination that leads to greater political participation, and not the other way around. We cannot verify this assumption within the context of this project, and it could be reasonably posited that through engagement in political activities, ethnic minorities become more aware of discriminatory practices in their environment. This is a limitation every other study on the topic of the relationship between experiences of discrimination and political participation has confronted; more broadly, it is a limitation faced by most studies of public opinion and political behavior that rely on cross-sectional data. Accordingly, future research could employ panel data to corroborate the findings of this study while simultaneously addressing the matter of causal direction.

## Appendix

**Appendix.** Construction of variables.

Participation in protest activities	Three dichotomous variables indicating whether or not over the last 12 months respondent has taken part in each of: 1. Boycotting 2. Signing a petition 2. Demonstrating in the street
Age	Age in years
Immigrant	1 = born outside Canada, 0 = born in Canada
Women	1= female, 0 = male
Education (highest degree completed)	Reference category = completed high school; Postsecondary = completed CEGEP (college); University = completed undergraduate degree; Postgraduate university = completed postgraduate degree
Unemployed	1 = unemployed, 0 = all others
Province of residence	Indicates whether respondents are from Quebec, Ontario, Alberta or British Columbia.
Ethnicity	Indicates the ethnicity of respondents.
Intragroup contact	0 to 3 scale indicating the extent to which respondent spends time with people of his/her own ethnic background – aside from their families.
Interest in politics	0 to 10 scale indicating the level of interest in politics. Scale combines responses to questions about interest in provincial politics and federal politics.
General social network	0 to 1 scale indicating the frequency at which respondents have contact: (a) with neighbours; (b) with colleagues outside work; and (c) with friends.

## Notes

1. At the same time, recent years have been marked by growing public awareness of discrimination and racism, notably through the mobilization of the Black Lives Matter movement and public discussions about systemic discrimination.

2. Wrinkle et al. (1996), however does not observe such a relationship among Cubans in the United States.

3. The evidence concerning the relationship between discrimination and voting tends to suggest a negative relationship in the United Kingdom (Martin, 2016), in the United States (Valdez, 2011) and in the province of Quebec in Canada (Bilodeau, 2017). Other studies in Canada, however, observe no relationship between discrimination and voting (Reitz and Banerjee, 2007; Tossutti, 2007). Another body of research focuses on the relationship between experiences of discrimination and ethnic minorities’ sense of belonging to the national community. Ethnic minorities who report discrimination tend to express a weaker attachment to the national community in which they find themselves as well as being less likely to identify within that community (Bilodeau, 2017; Döring, 2007; Jasinskaja-Lahti et al., 2009; Maxwell, 2006; Ono, 2002; Reitz and Banerjee, 2007).

4. Unfortunately, we do not have access to indicators of participation in the ethnic community. We do have an indicator of frequency of contact with people of the same ethnic background but this is not exactly the same thing as engagement in ethnic community and, as explained below, we use this variable for another purpose.

5. The sample was stratified by province with about 400 visible minority respondents in each province. The 25-minute online survey was commissioned by the authors (Bilodeau et al., 2014). Respondents received an e-mail invitation to participate in the survey. Each invitation email contained a unique link – Uniform Resource Locator (URL) – that could only be used once. Respondents were all members of the Léger Internet panel of more than 400,000 people currently living in Canada who were recruited randomly over the phone (61%) or through various other means. Léger’s annual recruitment rate for the panel is approximately 15,000 new members a year, while about 10,000 to 12,000 panelists are removed from the panel or opt out each year. Panelists are rewarded for their participation over time with a series of financial incentives. No specific response rate can be calculated for an online survey because, unlike telephone surveys, it is not possible to evaluate whether people refused to participate or did not read or receive the invitation. For the visible minority sample, 13,549 invitations were sent and 1647 respondents (12%) completed the survey. The data are weighted to be representative of the socio-demographic characteristics (sex, age, education, mother tongue and ethnicity) of each province, using the 2011 National Household Survey as the point of reference. The data are also weighted to ensure the proportional representation of each provincial sample according to its demographic weight within Canada. The survey was offered in English and in French.

6. Data used for this study did not include any information about respondents’ perception that their own ethnic group experiences discrimination.

7. In addition, ‘don’t know’ and ‘refuse to answer’ were offered as choices; between 5% and 7% of respondents provided one of these two answers to any single domain of discrimination.

8. The Pearson correlation coefficient between two indices of discriminatory experiences is .72.

9. We also analyzed the moderating effect of intragroup contact, looking at the *public sphere* and *private sphere* discrimination separately. The results provide no statistically significant evidence of a moderating effect when we examine each form of protest participation separately. However, our analyses using the participation index reveal a statistically significant moderating effect of intragroup contact, but only when discrimination takes place in the public sphere.

10. Arguably, general social contact could equally serve as a facilitator of political mobilization for visible minorities who experience discrimination. To test this possibility, we performed additional analyses on the interaction between discrimination and general social contact. We performed those analyses only for visible minorities who have limited or no contact with people from their own ethnic background, because our measurement of general social contact was otherwise likely to overlap with our measure of intragroup contact, given the wording of our items. The results provide no evidence of a mobilizing effect of general social contact for minorities experiencing discrimination. We did observe a significant interaction effect between general social contact and discrimination on participation in boycotts, but the effect was negative, suggesting that minorities reporting discrimination who also have a dense social network are less likely to engage in this form of political activity. Results not presented.
